# Phototherapy Induced Hypocalcemia in Term and Preterm Neonates with Hyperbilirubinemia: A Cross-Sectional Study

**DOI:** 10.31729/jnma.v63i290.9208

**Published:** 2025-09-01

**Authors:** Drishti Poudel, Sunil Tamang, Jamuna Neupane, Ramchandra Bastola, Yagya Raj Sigdel, Bharat Chand, Nabin Raj Gautam

**Affiliations:** 1 Department of Pediatrics, Nepal APF Hospital, Balambu, Kathmandu, Nepal; 2 Kanti Children's Hospital, Maharajgunj, Kathmandu, Nepal.; 3 Department of Pediatrics, Pokhara Academy of Health Sciences, Pokhara, Kaski, Nepal; 4 Green Pastures Hospital, Pokhara, Kaski, Nepal

**Keywords:** *hypocalcemia*, *neonatal hyperbilirubinemia*, *phototherapy*

## Abstract

**Introduction:**

Neonatal jaundice is a common condition affecting 60% of term and 80% of preterm neonates within the first week of life. Phototherapy, though the standard treatment for neonatal hyperbilirubinemia, may cause complications such as hypocalcemia, a significant but lesser-known side effect. This study compared phototherapy-induced hypocalcemia in late preterm and term neonates with unconjugated hyperbilirubinemia, after 48 hours of phototherapy.

**Methods:**

This hospital-based descriptive cross-sectional study was conducted in the Neonatal Intensive Care Unit of a tertiary care Hospital from 16th August 2022 to 10th July 2023. A total of 150 neonates, both term and preterm, with hyperbilirubinemia requiring at least 48 hours of phototherapy were enrolled. After ethical approval and informed consent, data were collected using a structured questionnaire and analyzed using IBM SPSS Statistics (version 23.0).

**Results:**

After 48 hours of phototherapy, calcium decreased in both groups. In term neonates, it decreased from 9.44±0.59 to 8.57±0.73 mg/dL, mean change was 0.87 mg/dL (95% CI 0.77-0.97) and in preterm neonates from 9.10±0.43 to 7.94±0.79 mg/dL, mean change was 1.16 mg/dL (95% CI 1.01-1.31). The between group difference was 0.29 mg/dL (95% CI 0.11-0.47), Cohen’s d = 0.55. Hypocalcemia occurred in 19 (21.30%) term and 21 (34.40%) preterm neonates (RR 1.61, 95% CI 0.95-2.73; RD 13.1%, 95% CI 7.2% to 32.8%).

**Conclusions:**

There is a reduction in serum calcium levels in both term and preterm neonates after phototherapy. While exploratory analyses suggested a higher risk of hypocalcemia in preterm neonates, subgroup comparisons were underpowered and should be interpreted cautiously.

## INTRODUCTION

Neonatal jaundice is a common condition in newborns affecting 60% of term and 80% of preterm in the first week of life.^1^ Phototherapy is one of the most frequently used therapeutic interventions in neonatal care, being the treatment of choice for neonatal unconjugated hyperbilirubinemia.^2^ While considered safe, phototherapy can lead to various complications, including skin tanning, skin rash, dehydration, hyperthermia, retinal damage, watery diarrhea, low platelet count, increased red cell osmotic fragility, riboflavin deficiency, DNA strand breaks, and bronze baby syndrome.^3^ Hypocalcemia is a lesser-known but significant complication induced by phototherapy, and the risk of hypocalcemia in preterm neonates is higher than in term neonates.^2^ Thus, this study was done to compare the occurrence of phototherapy-induced hypocalcemia between late preterm neonates and term neonates with unconjugated hyperbilirubinemia, after 48 hours of continuous phototherapy. The findings may help us to predict phototherapy-induced hypocalcemia and support timely interventions.

## METHODS

This was a hospital-based descriptive cross-sectional study conducted in Neonatal Intensive Care Unit of Pokhara Academy of Health Sciences (PoAHS), Pokhara, Kaski, a tertiary care hospital in Nepal from 16th August 2022 to 10th July 2023.

Ethical approval was obtained from the Institutional Review Committee of PoAHS (Reference number: 105/079).

The inclusion criteria were term neonates (≥37 weeks gestation) and late preterm neonates (34-36 weeks gestation) with unconjugated hyperbilirubinemia requiring phototherapy, as per American Academy of Pediatrics (AAP) 2004 guidelines.^4^ Neonates were excluded if they had hypocalcemia before phototherapy, a 5 minute APGAR score <7, were born to diabetic mothers, required exchange transfusion, had culture-proven sepsis, congenital anomalies, or if the mother was on anticonvulsant therapy. Neonates not requiring 48 hours of phototherapy or whose parents did not provide consent were also excluded.

Sample size was calculated using the formula for comparing the means of two independent groups:

n = 2(Z_α/2_ + Z_1-β_)^2^ σP^2^/Δ^2^

Where, Z_α/2_ = 1.96 (for 95% confidence level)

  Z_1-β_ = 0.84 (for 80% power)

  σ_p_ = pooled standard deviation

  Δ = expected difference between two means

  From a previous study, the mean serum calcium after phototherapy in term neonates was 8.17±0.78 and in preterm neonates was 7.39±0.53.^5^

n = 12

Thus, the minimum required sample size was 24 neonates (12 in each group).

However, since the prevalence of hypocalcemia after phototherapy is reported to be high, and considering feasibility, the sample size was alternatively calculated using the single proportion formula:

n = Z^2^ × p(1-p)/d^2^

n = required sample size

Z = standard normal deviation for a 95% confidence level (1.96)

p = estimated proportion in the population (9.6%)^6^

d = the margin of error at 5% (0.05)

n = 134

After adjusting 10% non-response rate, sample size is 147 ; 150. In a prospective cross-sectional study done by Goyal et al., overall incidence of hypocalcemia after phototherapy was found to be 9.6%.^6^

After obtaining the informed written consent from the parents, neonates up to 7 days of life admitted for phototherapy for unconjugated hyperbilirubinemia were enrolled consecutively until the required sample size of 150 was reached.

Data were collected using a structured proforma that included demographic details such as sex and gestational age, the latter assessed using New Ballard scoring.^7^ Patient charts were reviewed for birth weight, APGAR score, gestational and intrapartum history, postnatal events, breastfeeding adequacy, and feeding difficulties. A detailed clinical examination was also performed. Neonates with hyperbilirubinemia were managed as per the AAP 2004 guidelines.^4^ Double-surface phototherapy was administered using Phototherapy equipment from MTTS Co., Ltd. emitting blue light (wavelength 455-470 nm) with an average irradiance of 42-45 μ/cm^2^/nm and standard protocols were followed.^8^

Total serum bilirubin level and total calcium level were measured before and after 48 hours of phototherapy. In this study, hypocalcemia was defined as total serum calcium of <8 mg/dL in term and < 7 mg/dL in preterm neonates.^9^ Neonates were also clinically assessed for features of hypocalcemia. Data were analyzed using IBM SPSS Statistics (version 23.0). Frequency and percentages were calculated for categorical variables while mean and standard deviation were calculated for continuous variables. Comparisons between term and preterm group were exploratory and expressed using effect sizes with 95% confidence intervals (mean differences with Cohen’s d for continuous outcomes, and risk ratios (RR) with risk differences (RD) for binary outcomes). Tests were performed at 95% confidence interval (CI).

## RESULTS

A total of 150 neonates with hyperbilirubinemia were enrolled in the study, comprising 89 (59.30%) term and 61 (40.70%) preterm neonates. Among them, 86 (57.30%) were male while 64 (42.70%) were female. Male-to-female ratio in term and preterm neonates was 1.02:1 and 2.05:1 respectively. The mean gestational age was 39.50±1.20 weeks for term neonates and 35.60±0.70 weeks for preterm neonates. Mean birth weight in term and preterm neonates was 3174.10±444.80 gm and 2366.50±456.70 gm respectively.

**Table 1:** Baseline Characteristics of the Study Population (n=150).

Variable	Term Neonates (n=89)	Preterm Neonates (n=61)
Male: Female Ratio	1.02:1	2.05:1
Gestational Age (weeks), Mean±SD	39.5±1.20	35.6±0.70
Birth Weight (g),	3174.1±444.80	2366.5±456.70
Exclusive Breastfeeding	35	35
Hours of life at initiation of phototherapy	69.37±19.80	67.15±16.40
Serum Bilirubin before phototherapy, (mg/dL)	18.46±2.05	16.60±1.61
Serum Bilirubin after phototherapy (mg/dL)	12.7±2.14	11.37±1.58
Duration of phototherapy (hours)	53.31±4.80	52.02±4.30

**Table 2:** Serum Calcium Before and After Phototherapy between Preterm and Term Neonates (n=150).

Group	Serum Calcium before phototherapy (mg/dL), Mean±SD	Serum Calcium after photo therapy (mg/dL), Mean±SD	Mean Difference (mg/dL)	95% CI for mean change
Term	9.44±0.59	8.57±0.73	0.87±0.49	0.77-0.97
Preterm	9.10±0.43	7.94±0.79	1.16±0.58	1.01-1.31

The mean total serum bilirubin (TSB) before phototherapy was 18.46±2.050 mg/dL in term neonates and 16.60±1.61 mg/dL in preterm neonates. After 48 hours of phototherapy, TSB was 12.70±2.14 mg/dL and 11.37±1.58 mg/dL in term and preterm neonates respectively. The duration of phototherapy in term neonates was 5 3.31 ±4.80 hours and in preterm neonates was 52.02±4.30 hours.

This study demonstrated a reduction in serum calcium levels following phototherapy in both term and preterm neonates (Table 2). In term neonates, serum calcium was 9.44±0.59 mg/ dL to 8.57±0.73 mg/dL before and after phototherapy and mean change of 0.87 mg/dL (95% CI 0.77-0.97). In preterm neonates, serum calcium was 9.1±0.43 mg/dL and 7.94±0.79 mg/dL before and after phototherapy and mean change was 1.16 mg/dL (95% CI 1.01-1.31). The difference in mean changes between two groups was 0.29 mg/dL (95% CI 0.11-0.47), corresponding to a Cohen’s d = 0.55 (moderate). The post-hoc power analysis indicated that this comparison had ;91% power, suggesting that the study was adequately powered for this observed effect.

It was observed that 19 (21.30%) neonates of term group and 21 (34.40%) of preterm group developed hypocalcemia after phototherapy, corresponding to a risk ratio of1.61 (95% CI 0.952.73) and a risk difference of 13.1% (95% CI -7.20% to 32.80%). A post-hoc analysis indicated that the power (~42%) was not adequate to detect this proportion difference and the results are interpreted as exploratory. Among neonates with hypocalcemia, jitteriness was observed in 10 (47.60%) hypocalcemic preterm neonates and 2 (10.50%) term neonates. None of the neonates developed severe clinical manifestations, such as convulsions or apnea.

**Table 3:** Hypocalcemia betweenPreterm and TermNeonates After Phototherapy (n=150).

Group	Hypocalcemia n(%)	Normal Calcium n(%)
Term (n=89)	19(21.30%)	70(78.70%)
Preterm (n=61)	21(34.40%)	40(65.57%)
Effect estimates: RR = 1.61 (95% CI 0.95-2.73); RD = 13.1% (95% CI -7.2% to 32.8%).		

**Table 4:** Comparison of Clinical Features of Hypocalcemia between Preterm and Term Neonates (n=40).

Clinical features of hypocalcemia	Preterm n(%)	Term n(%)
Jitteriness n(%)	10(47.60%)	2(10.50%)
No symptoms n(%)	11(52.40%)	17(89.50%)

Post-hoc power for this comparison was ~42%.

There were 10 (47.60%) preterms with symptoms of jitteriness while 2 (10.50%) term neonates had jitteriness.

## DISCUSSION

Neonatal jaundice is a common condition in the first week of life and is also a common cause of hospital admission.^10^ Phototherapy is considered safe and effective treatment for treating neonatal hyperbilirubinemia and the long-term side effects of phototherapy are minimal, absent, or unrecognized.^11^ This study evaluated the effect of phototherapy on serum calcium levels in both term and preterm neonates, as hypocalcemia is one of the potential complications of phototherapy among others.^12^

In this study, 150 neonates were included with a higher proportion of term neonates (59.30%) compared to preterm (40.70%). This distribution is because preterm neonates who were sick were excluded from the study. Among 150 neonates, 57.3% were male similar to the findings by Goyal et al. in India and Shrestha et al. in Nepal with higher incidence among male in the study population.^6,13^ This might be because male infants are at higher risk of developing significant neonatal jaundice than female infants.^14^

The mean gestational age of this study group for preterm and term neonates were 35.6±0.7 weeks and 39.5±1.2 weeks respectively which was comparable to study of similar settings of Basnet et al. in Nepal,^15^ likely because of shared ethnicity, socio-economic and environmental factors and similar health care services. Exclusive breastfeeding among children aged 0-5 months according to NDHS 2022 is 56%.^16^ However among the neonates in this study group only 39.3 % of term neonates and 57.3 % of preterm neonates were exclusively breastfed. This may be due to higher LSCS rates (35.3%) where mothers might not be physically ready for exclusive breastfeeding. Studies have also shown that babies born via caesarean section had significantly lower odds of exclusive breastfeeding.^17^

In the study, there was decrease in serum calcium levels after 48 hours of phototherapy in both term (mean difference: 0.88±0.49 mg/dL) and preterm neonates (1.16±0.58 mg/dL) with greater decline noted in preterm neonates. These findings were comparable to study done by Basnet et al., the mean difference in preterm and term neonates was 0.705±0.861 mg/ dl and 0.915±0.779 mg/dL respectively.^15^ Similar to our findings of a decline in total serum calcium after phototherapy, Goyal et al. also observed a statistically significant reduction in both total and ionized calcium after phototherapy in both term and preterm neonates compared to control. The reduction was numerically slightly greater in preterm neonates (0.98 mg/dL) than in term neonates (0.92 mg/dL).^6^ The greater vulnerability of preterm neonates might be because of immature calcium homeostasis mechanisms.^18^

The incidence of phototherapy induced hypocalcemia in neonates varies widely across the literature. In term neonates, it ranged from as low as 3-9% to as high as 66.6%, while in preterm neonates, the incidence has reached up to 80% in some studies.^6,11,13,19-23^ In contrast, Basnet et al. reported hypocalcemia in only 5.3% of term neonates and none among preterms.^15^ In our study, hypocalcemia was observed in 21.3% of term and 34.4% of preterm neonates after 48 hours of phototherapy, which aligns with findings by Jain et al.^19^ and Gusain et al.^24^, who also measured serum calcium following a similar phototherapy duration. Shrestha et al. reported comparable results, although calcium levels were assessed only after phototherapy cessation.^13^ The wide variation in hypocalcemia incidence may be attributed to methodological differences among studies, such as the timing of calcium measurement and whether total or ionized calcium was assessed. For example, Yadav et al. and Jain et al. evaluated ionized calcium,^19,21^ whereas Chandrasekhar et al. and Gusain et al. focused on total serum calcium^20,24^ Goyal et al. uniquely assessed both.^6^ Since ionized calcium is the physiologically active form and tightly regulated by homeostatic mechanisms, discrepancies may arise when relying solely on total serum calcium, which is influenced by albumin levels and pH.^25^ It is unknown if other elements, such as the type of fluorescent tube utilized, the technique employed to estimate the serum calcium level, etc., also had an impact on the outcomes.

In our study, symptomatic hypocalcemia was observed in 47.6% of preterm and 10.5 % term neonates, suggesting increased susceptibility in preterm neonates as noted by Arora et al. who reported symptomatic hypocalcemia more in preterm than term.^26^ Ionized Calcium plays a vital role in numerous biochemical processes, such as blood clotting, neuromuscular excitability, cell membrane integrity, and many cellular enzymatic activities.^27^

Common clinical symptoms of hypocalcemia are apnea, seizures, jitteriness, increased extensor tone, clonus, hyperreflexia, stridor, etc.^18^ In this study, all of the newborns that are symptomatic, jitteriness was the sole clinical manifestation aligning with known hypocalcemia related neuromuscular excitability. Consistent with our findings, Yadav et al. reported jitteriness, irritability, and lethargy as the primary manifestations of hypocalcemia but none had a convulsion.^21^ In study done by Shrestha et al. and Goyal et al., significantly lower rates (2.3% and 2.86%, respectively)^6,13^ of symptomatic hypocalcemia was reported while Arora et al., Gusain et al., and Yadav et al. demonstrated findings consistent with ours.^21,24,26^ The variation in symptomatic hypocalcemia among different studies may be attributed to differences in neonatal characteristics, clinical recognition of subtle and overlapping symptoms like jitteriness and irritability and the proportion of preterm neonates who are physiologically more vulnerable to hypocalcemia due to immature parathyroid function and lower calcium stores.

The results of this study make it apparent that phototherapy causes hypocalcemia either symptomatic or asymptomatic. Future research will need to determine the precise cause; however low birth weight and preterm newborns had a higher incidence of hypocalcemia. While various mechanisms by which phototherapy induces hypocalcemia are proposed, exact cause remains uncertain. These results underscore the need for further research to explore effect of calcium regulation in neonates receiving phototherapy and to identify potential preventive or therapeutic strategies.

Notably, the post-hoc analysis showed that our study was adequately powered to detect the observed difference in mean calcium change (power ;91%). However, power was limited for detecting differences in hypocalcemia proportions (;42%).

The strengths of this study include a relatively large sample size, standardized methodology, and assessment of both biochemical and clinical features of hypocalcemia. However, some limitations must be noted. Being a single center cross-sectional study, the generalisability of results to other populations is limited. Only total serum calcium was measured, which may not fully reflect the physiologically active ionized calcium. Also, the sample size was calculated based on prevalence estimates, and subgroup analyses between term and preterm neonates were not pre-specified for hypothesis testing. Therefore, comparisons between groups should be interpreted as exploratory. These limitations should be taken into account when interpreting the study results.

## CONCLUSION

There is significant decrease in serum calcium level after 48 hours of continuous phototherapy in both term and late preterm neonates. 1 in 3 preterm neonates and 1 in 5 term neonates developed hypocalcemia after 48 hours of phototherapy. Jitteriness was the only clinical feature of hypocalcemia, noted more frequently in preterm neonates undergoing phototherapy for hyperbilirubinemia.

## Data Availability

The data are available from the corresponding author upon reasonable request.

## References

[1] National Institute for Health and Care Excellence (2014). Jaundice in Newborn Babies Under 28 Days.. NICE Qual Stand..

[2] Sethi H, Saili A, Dutta AK (1993). Phototherapy Induced Hypocalcemia.. Indian Pediatr..

[3] Xiong T, Qu Y, Cambier S, Mu D (2011). The Side Effects of Phototherapy for Neonatal Jaundice: What Do We Know? What Should We Do?. Eur J Pediatr..

[4] American Academy of Pediatrics Subcommittee on Hyperbilirubinemia (2004). Management of Hyperbilirubinemia in the Newborn Infant 35 or More Weeks of Gestation.. Pediatrics..

[5] Jha CB, Rimal HS, Subedi RB (2022). Phototherapy Induced Hypocalcemia in Icteric Newborns Attending Birat Medical College Teaching Hospital, Morang, Nepal.. Journal of Chitwan Medical College..

[6] Goyal M, Sharma R, Dabi DR (2018). Phototherapy induced hypocalcemia in neonates: A case.. Indian Journal of Child Health..

[7] Ballard JL, Khoury JC, Wedig K, Wang L, Eilers-Walsman BL, Lipp R (1991). New Ballard Score, Expanded to Include Extremely Premature Infants.. J Pediatr..

[8] MTTS-Asia (2020). Firefly Phototherapy.. https://www.mtts-asia.com/firefly-phototherapy/.

[9] Namgung R, Tsang RC, Oh W, Baum M (2019). Perinatal Calcium and Phosphorus Metabolism.. Nephrology and Fluid/Electrolyte Physiology..

[10] Burke BL, Robbins JM, Bird TM, Hobbs CA, Nesmith C, Tilford JM (2009). Trends in Hospitalizations for Neonatal Jaundice and Kernicterus in the United States, 1988-2005.. Pediatrics..

[11] Karamifar H, Pishva N, Amirhakimi GH (2002). Prevalence of Phototherapy-Induced Hypocalcemia.. Iran J Med Sci..

[12] Alizadeh-Taheri P, Sajjadian N, Eivazzadeh B (2013). Prevalence of Phototherapy Induced Hypocalcemia in Term Neonate.. Iran J Pediatr..

[13] Shrestha S, Budhathoki S, Sanjel S, Sindan N, Kayastha N, Shrestha A (2021). Effect of Phototherapy on Serum Calcium Level in Neonatal Hyperbilirubinemia.. J Karnali Acad Health Sci..

[14] Scrafford CG, Mullany LC, Katz J, Khatry SK, LeClerq SC, Darmstadt GL (2013). Incidence and Risk Factors for Neonatal Jaundice Among Newborns in Southern Nepal.. Trop Med Int Health..

[15] Basnet S, Bhatta Gauchan E, Bhatta M, Thapa R (2022). Phototherapy Induced Hypocalcemia in Neonatal Hyperbilirubinemia and Correlation of Hypocalcemia With the Duration of Phototherapy.. Int J Contemp Pediatr..

[16] Ministry of Health and Population (NP), New ERA, ICF (2022). Nepal Demographic and Health Survey 2022: Key Indicators Report.. https://dhsprogram.com/pubs/pdf/PR142/PR142.pdf.

[17] Show KL, Jampathong N, Aung PL, Win KM, Ngamjarus C, Pattanittum P (2023). Does Caesarean Section Have an Impact on Exclusive Breastfeeding? Evidence From Four Southeast Asian Countries.. BMC Pregnancy Childbirth..

[18] Eichenwald EC, Hansen AR, Martin CR, Stark AR (2021). Cloherty and Stark's Manual of Neonatal Care..

[19] Jain BK, Singh H, Singh D, Toor NS (1998). Phototherapy-Induced Hypocalcemia.. Indian Pediatr..

[20] Chandrashekar C (2014). Effect of Duration of Phototherapy on Serum Calcium Level in Newborns With Neonatal Jaundice.. Pediatr Rev Int J Pediatr Res..

[21] Yadav RK, Sethi RS, Sethi AS, Kumar L, Chaurasia S (2012). The Evaluation of Effect of Phototherapy on Serum Calcium Level.. Peoples J Sci Res..

[22] Gheshmi AN, Naderi S, Homayrani E, Safari B (2015). Prevalence of Hypocalcemia After Phototherapy Among Neonates Who Underwent Phototherapy in Koodakan Hospital in Bandar Abbas in 2013.. Electron Physician..

[23] Rozario CI, Pillai PS, TR (2017). Effect of Phototherapy on Serum Calcium Level in Term Newborns.. Int J Contemp Pediatr..

[24] Gusain P, Gupta VK, Gupta S, Natani BS, Agrawal S, Yadav N (2017). Comparative Analysis of Symptomatic Hypocalcemia in Term and Preterm Neonates Undergoing Continuous Phototherapy.. IOSR J Dent Med Sci..

[25] Albert SG, Isbell TS (2023). Reconsideration of "Albumin Corrected Total Calcium" Determinations: Potential Errors in the Clinical Management of Disorders of Calcium Metabolism.. Clin Chim Acta..

[26] Arora S, Narang G, Singh G (2014). Serum Calcium Levels in Preterm and Term Neonates on Phototherapy.. J Nepal Paediatr Soc..

[27] Hall JE, Hall ME (2020). Guyton and Hall Textbook of Medical Physiology..

